# Meteorological Register for March, 1817

**Published:** 1817-06

**Authors:** John Bailey

**Affiliations:** Surgeon, &c.


### Meteorological Register for March, 1817.

*Kept at Harwich, in Essex.*

By JOHN BAILEY, Surgeon, &c.

| Moon's Age. | | Atmospherical Pressure. Morn. | Ev. | Temp. | Wind. | Remarks. | General Statement of Diseases. |
|---|---|---|---|---|---|---|---|
| | 1 | 29 6 | 29 5 | 52 | W | Windy | |
| | 2 | 6½ | 6½ | 48 | W | Rain and wind | |
| ○ | 3 | 3 | | 48 | SW | Rain | |
| | 4 | | 1½ | 43 | NW | Rain, G. of W. | |
| | 5 | 2 | | 44 | W | ditto | |
| | 6 | 28 8 | 29 1 | 40 | NW | Rain and snow | |
| | 7 | 29 1 | 1 | 37 | NW | Fine | |
| | 8 | 6 | 2 | 35 | NW | Rain and snow | |
| | 9 | 3½ | 6 | 34 | NW | Snow | |
| ☾ | 10 | 8 | 30 ½ | 40 | N | Fine | |
| | 11 | 30 ½ | | 54 | W | ditto | |
| | 12 | 29 9 | 29 8 | 55 | NW | Rain G. of W. | |
| | 13 | 8½ | 30 | 56 | W | Rain | |
| | 14 | 30 1 | ½ | 44 | SE | Fine | |
| | 15 | 2 | 2 | 44 | S | ditto | |
| | 16 | | | 43 | SE | ditto | |
| ● | 17 | | | 49 | SE | Foggy | |
| | 18 | | | 50 | SE | Thick Fog | |
| | 19 | 29 3 | 29 7 | 47 | W | Rain | |
| | 20 | 7 | | 31 | N | Snow | |
| | 21 | ½ | 8 | 30 | N | Deep Snow | |
| | 22 | 8 | 9 | 42 | SE | Fine | |
| | 23 | 8 | 8 | 43 | SE | Rain | |
| | 24 | 8 | 6 | 43 | SE | Fine | |
| | 25 | 6½ | 6½ | 52 | W | Rain and wind | |
| ☽ | 26 | 6 | 7 | 51 | W | Windy | |
| | 27 | 8½ | 30 | 45 | NW | Rain | |
| | 28 | 9 | 9 | 45 | SW | ditto | |
| | 29 | 7 | 8 | 55 | W | | |
| | 30 | 9 | 9 | 55 | W | | |
| | 31 | 30 1 | 30 1 | 48 | NW | Rain | |

With the exception of two cases of parotidis, there have been no occurrences of atmospherical disorder. The windy month of March has this year run its course with unusual mildness; little wet has fallen to the earth; mild and brisk winds have speedily dried it away, and disorder has had no opportunity of fixing its seat amongst us.

